# Decreasing Medication Errors at Discharge: A Quality Improvement Project

**DOI:** 10.1097/pq9.0000000000000599

**Published:** 2022-10-03

**Authors:** Carolyn Walker, Oluwafemi Ojo, Matthew Garcia, Jennifer Hutchison, Dillon Lesak, Laura Calvo, Jordi Friese, Candace Garza, Sally Barman, Ernest Buck, Lee Budin

**Affiliations:** From the Medical Education Department and Quality and Patient Safety Department, Driscoll Children’s Hospital, Corpus Christi, Tex.

## Background:

Medication reconciliation is a part of a National Patient Safety Goal, and suboptimal medication reconciliation at discharge has been found to be associated with 28% of potentially avoidable readmissions within 30 days (Tong et al, 2017). As part of a hospital-wide initiative to improve the clarity and completeness of medications listed on the after visit summary (AVS), we noted that at our institution, PRN (as needed) medication errors made up 27% of the medication errors on the AVS. The aim of our project was to increase the percentage of PRN medications that met our medication reconciliation bundle criteria from 56% to 64% by July 31, 2021 and increase it by 3% every 3 months until July 31, 2022, with a goal to sustain at 75% through the following year, for all inpatient AVS discharges that are audited.

## Methods:

**Fig. 1.**
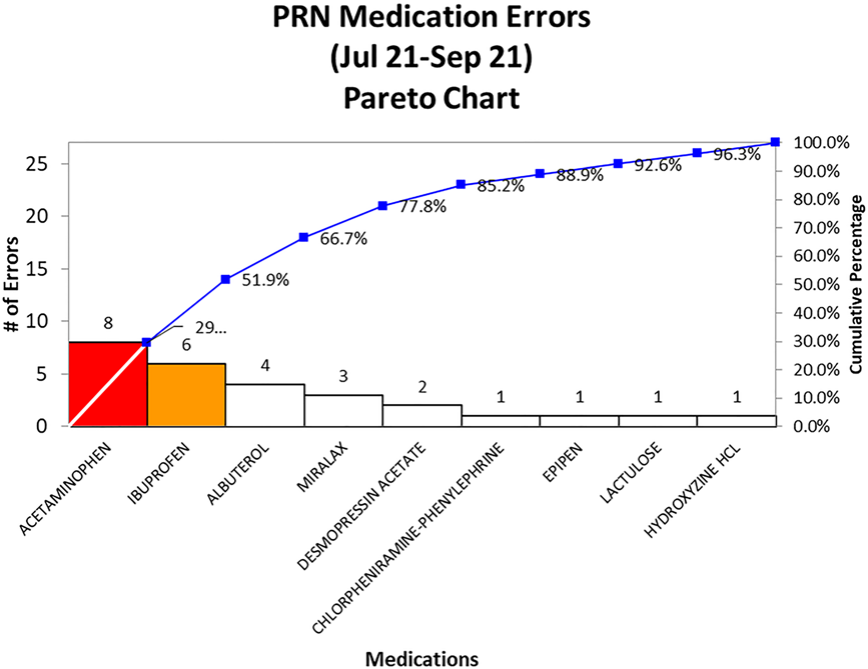
PRN medication errors Pareto chart.

Interventions were targeted toward our top error-prone medications based on a Pareto chart (Fig. 1). Interventions included implementation of a discharge order set for patients needing dual analgesics with prefilled instructions ensuring the 5 rights and alternation instructions, standardized MiraLax administration instructions, timely completion of admission medication reconciliation, and optimizing medication prescription instructions in the electronic medical record.

## Results:

**Fig. 2.**
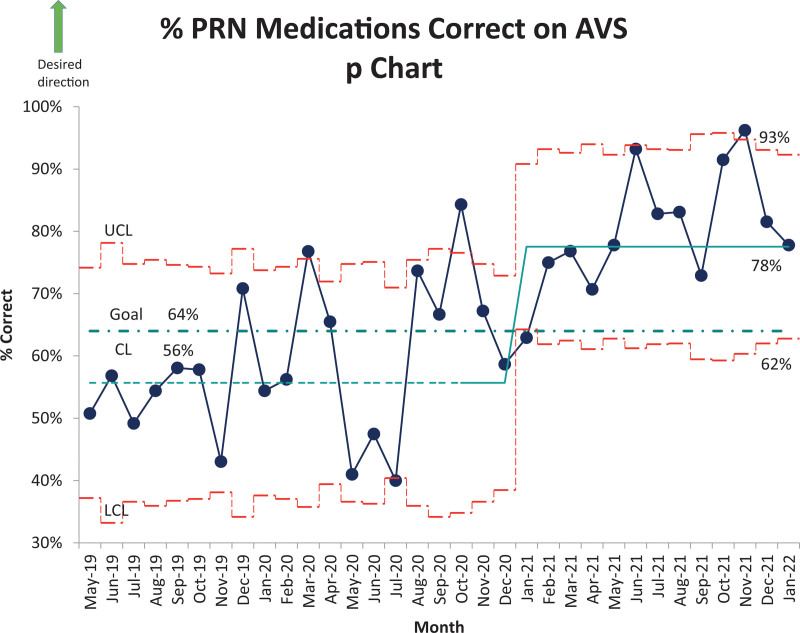
Outcome metric: % PRN medications correct on AVS p chart.

After multiple Plan-Do-Study-Act (PDSA) cycles over 5 months, we had a shift from a baseline of 56% to 78% (Fig. 2). The 3 months ending July 2021 showed an average accuracy of 80%, which surpassed our goal of 64%.

## Conclusions:

We were able to improve our discharge medication reconciliation efforts for PRN medications beyond our predicted goal using QI methods and collaboration. Our next steps include sustaining current gains while implementing interventions on other PRN medications and outpatient scheduled medication.

The process measures showed that there was a reduction of 15 errors/50 audited charts to 0 errors for the analgesics. MiraLAX interventions also showed similar improvement from 80% to 90% accuracy. We saw an increase in the admission medication reconciliation rate from a baseline of 73% of patients to 96% by the end of the PDSA cycles.

